# Comparison of Transmissibility of Coronavirus Between Symptomatic and Asymptomatic Patients: Reanalysis of the Ningbo COVID-19 Data

**DOI:** 10.2196/19464

**Published:** 2020-05-28

**Authors:** Guosheng Yin, Huaqing Jin

**Affiliations:** 1 Department of Statistics and Actuarial Science The University of Hong Kong Hong Kong China

**Keywords:** asymptomatic case, close contact, coronavirus, COVID-19, Fisher exact test, transmission rate, transmission, virus, immunology, analysis

## Abstract

**Background:**

Since the outbreak of the novel coronavirus disease (COVID-19) in December 2019, the coronavirus has spread all over the world at an unprecedented rate. The transmissibility of the coronavirus from asymptomatic patients to healthy individuals has received enormous attention. An important study using COVID-19 data from the city of Ningbo, China, was carried out to estimate and compare the transmission rates of the coronavirus by the symptomatic and asymptomatic patients. However, in the original analysis, the usual chi-square tests were unduly used for some contingency tables with small cell counts including zero, which may violate the assumptions for the chi-square test.

**Objective:**

We reanalyze the data from the city of Ningbo with more appropriate statistical methods to draw more reliable and sound conclusions on the transmission rates of the coronavirus by the symptomatic and asymptomatic patients.

**Methods:**

We excluded the cases associated with the super-spreader and adopted a more appropriate statistical method, including the permutation test and the Fisher exact test, to reanalyze the COVID-19 data from the city of Ningbo.

**Results:**

After excluding the cases related to the super-spreader, the Fisher exact test yields a *P* value of .84, which indicates stronger evidence of no difference in the transmission rates compared with the original analysis. The odds ratio of the coronavirus transmission rates between the symptomatic and asymptomatic patients is 1.2 with a 95% confidence interval 0.5-2.8.

**Conclusions:**

Through a more in-depth and comprehensive statistical analysis of the Ningbo data, we concluded that there is no difference in the transmission rates of coronavirus between symptomatic and asymptomatic patients.

## Introduction

Since the outbreak of the novel coronavirus disease (COVID-19) in December 2019, the coronavirus has spread all over the world at an unprecedented rate. By May 21, 2020, more than 200 countries and territories have been affected by COVID-19, with a total of more than 5 million confirmed cases and over 330,000 deaths [1]. In addition, both the numbers of cases and deaths continue to climb up quickly. On March 11, 2020, COVID-19 was declared an international public health emergency by the World Health Organization [2]. Many countries have taken the most restrictive travel bans and quarantine policies in an attempt to stop the coronavirus from infecting their healthy populations. The worldwide economy has also been greatly set back.

During the disease incubation period, a percentage of coronavirus carriers may have no symptoms or minimal symptoms and thus often go undetected. These covert coronavirus carriers may not even be aware of the infection themselves but would be confirmed as positive cases if tested using the reverse transcriptase polymerase chain reaction (RT-PCR). If the percentage of asymptomatic carriers is large and if their transmissibility of coronavirus is as high as the symptomatic cases, this would pose a great threat to the public health worldwide. Therefore, it is critical to determine the percentage and the transmissibility of asymptomatic coronavirus carriers in the population.

There has been some work in the literature on the estimation of the asymptomatic proportion of COVID-19 cases. Based on the infected cases on the Diamond Princess cruise ship, the asymptomatic ratio was estimated to be 0.179 with a 95% Bayesian credible interval of 0.155-0.202 [3]. Another study [4] indicated that the asymptomatic ratio could be as high as 0.416 by using the information on Japanese nationals who were evacuated from Wuhan, China on charter flights. An analysis on the COVID-19 infected cases from Tibetan Autonomous Prefecture [5] found that the proportion of asymptomatic carriers was 0.217. In a study with 36 children with COVID-19 in Zhejiang, China [6], it was found that there were 10 asymptomatic cases out of 36 infections (27.7%). Another investigation in a skilled nursing facility in King County, Washington identified that, out of 48 residents that tested positive for COVID-19, 27 (56%) were asymptomatic at the time of testing [7]. The aforementioned studies indicate that the proportion of asymptomatic carriers in the total infected cases is considerably high, but the sample sizes of these studies are rather small.

There has been evidence for transmission of coronavirus from asymptomatic carriers. It was reported that the viral load detected in the asymptomatic patients was similar to that in the symptomatic patients, which suggests the potential transmissibility of asymptomatic carriers [8]. A familial cluster of 5 patients in Anyang, China demonstrated transmission of the coronavirus from an asymptomatic carrier with normal chest computed tomography but tested positive after all 5 contacted family members had shown symptoms and confirmed positive RT-PCR test results [9]. A similar case of the familial cluster of 5 members associated with COVID-19 in Luzhou, China also suggested that coronavirus can be transmitted by asymptomatic carriers [10]. Another example of coronavirus infection by an asymptomatic patient was a German case through the usual contact in business meetings [11]. Moreover, the mathematical model developed to estimate the basic reproductive number of COVID-19 and quantify the contribution of different transmission routes also indicated the transmissibility of the asymptomatic individuals [12]. In a study on a cluster of 22 close contacts of a male 22 years of age with COVID-19 [13], the asymptomatic patient showed the rapid human-to-human transmissibility. Via a detailed literature review conducted at the Centers for Disease Control and Prevention [14], it was demonstrated from the epidemiologic, virologic, and modeling studies that COVID-19 is transmittable by persons with presymptomatic or asymptomatic infection.

Chen et al [15] carried out an important study using the COVID-19 data from Ningbo, China to estimate the transmission rates of the coronavirus by the symptomatic and asymptomatic cases. The estimated transmission rates for the symptomatic and asymptomatic patients were 0.063 and 0.041, respectively, and the chi-square test yielded a *P* value of .29, which indicates that there is no statistically significant difference between the two transmission rates. They further investigated the transmission rates for different relationships and different types of contact with the infected patients including both symptomatic and asymptomatic cases. The conclusions were that there are statistically significant differences in the transmission rates across different relationships and different types of contact. As expected, the closer the contact is with the infected patients, the higher the chance of infection.

The following is the permutation test algorithm:


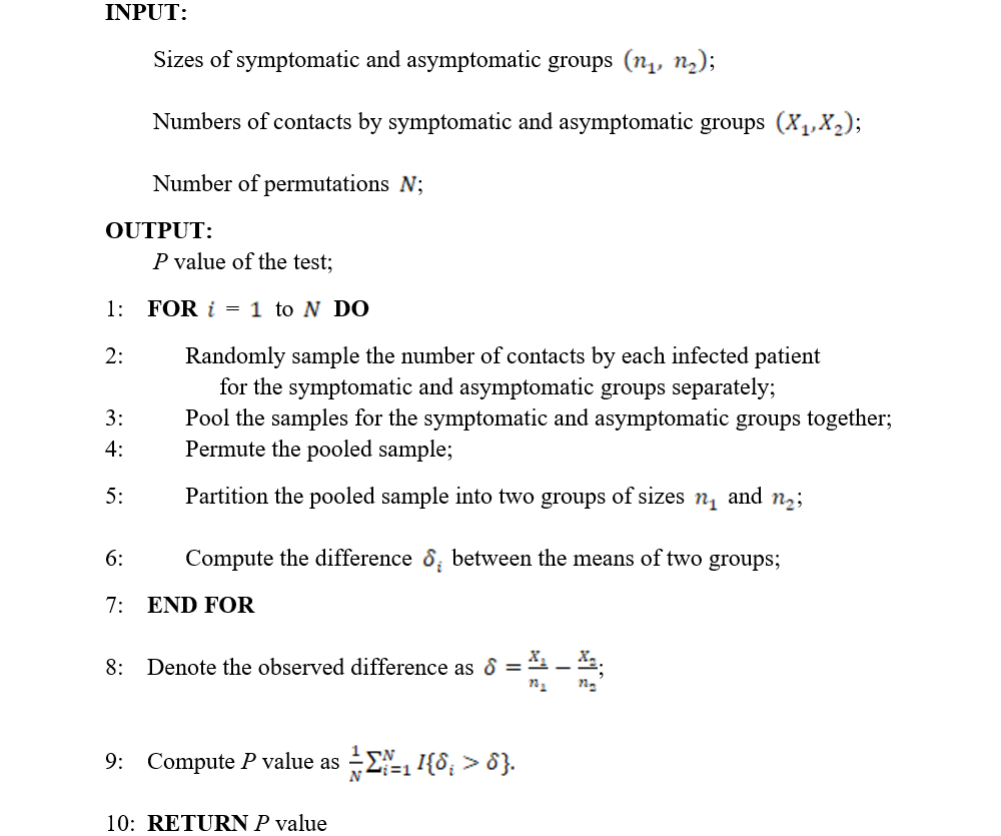


However, in their original statistical analysis [15], the chi-square tests were unduly used because the counts in some cells of the contingency tables were rather small and sometimes even zero, which violates the assumptions of a chi-square test and thus casts doubt on the validity of the hypothesis test. Moreover, when comparing the transmission rates of symptomatic and asymptomatic cases, Chen et al [15] included the cases associated with a super-spreader who mainly transmitted the disease in an air-conditioned bus and a Buddhism activity gathering. However, this may reduce the generalization of the findings, as the super-spreader should be regarded as an outlier and removed from the primary analysis.

## Methods

### Permutation Test

**Figure 1:**
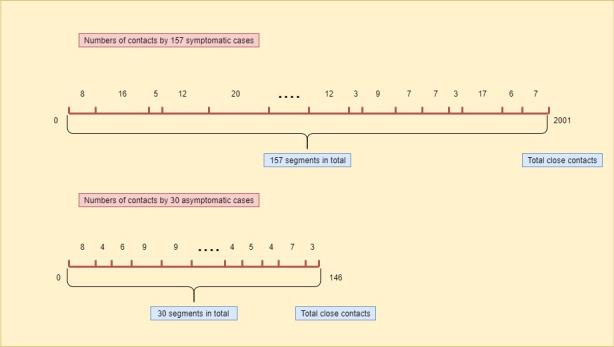
The diagram for the resampling step in the permutation test, where the lengths of segments are randomly generated corresponding to the number of close contacts for each individual patient.

We adopted a permutation test to determine the difference in the average numbers of contacts by the symptomatic and asymptomatic cases. The permutation test algorithm gives the details of the permutation test, and Figure 1 provides the diagram for the resampling step in the permutation test. Note that the permutation test requires no assumptions on the data, which simply permutes the data to simulate the null distribution.

### Fisher Exact Test

To allow for small cell counts including zero in the contingency table, the Fisher exact tests [16] were used to investigate the difference in the transmission rates between the symptomatic and asymptomatic patients wherever small cell counts were present (eg, less than 5 as a rule of thumb).

Without making any assumptions on the data, the Fisher exact test simply adopts the hypergeometric distribution to calculate the exact probability of the observed data in the table. For example, as shown in Table 1, suppose that there are *a* infected cases and *b* uninfected individuals in the close contacts of the symptomatic cases, while there are *c* infected cases and *d* uninfected individuals in the close contacts of the asymptomatic cases. The probability of observing such data is given by:


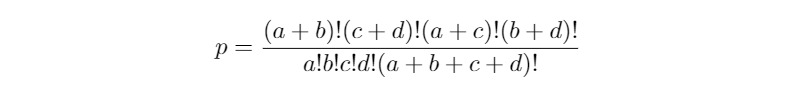


The *P* value of the Fisher exact test is calculated by summing up all the probabilities of obtaining data as or more extreme than the observed under the null hypothesis (ie, there is no difference between the two groups).

**Table 1:** A typical 2×2 contingency table.

Group	Infected	Uninfected
Number of close contacts of symptomatic cases	*a*	*b*
Number of close contacts of asymptomatic cases	*c*	*d*

### Odds Ratio and Related Confidence Intervals

To gain more insight into the Ningbo data, we further calculated the odds ratio between the symptomatic and asymptomatic groups as well as the corresponding confidence interval. For a 2×2 contingency table with cell counts (*a*, *b*, *c*, *d*) as shown in Table 1, the odds ratio is *ad*/*bc*, and the corresponding 95% confidence interval is given by:


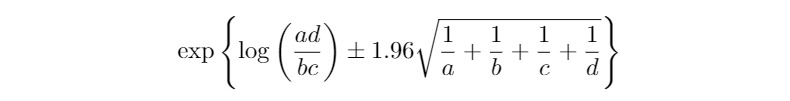


If the estimated transmission rate with sample size *n* is denoted by 
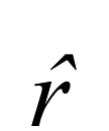
, the 95% confidence interval of the transmission rate is:

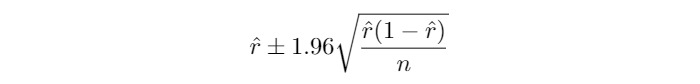



Both confidence intervals for the odds ratio and the transmission rate are based on normal approximation, and in the Ningbo data, the sample sizes for computation of these confidence intervals are reasonably large.

## Results

### Results of the Permutation Test

**Figure 2:**
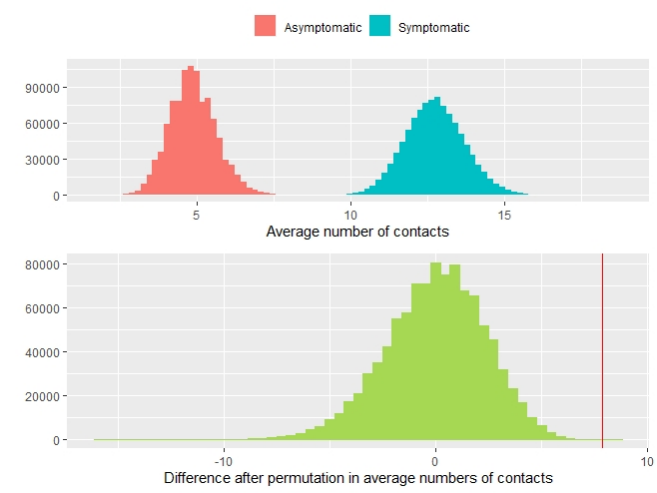
The histograms of the average numbers of contacts by the symptomatic and asymptomatic cases (top panel) and the difference after the permutation in the average numbers of contacts by the symptomatic and asymptomatic cases in the permutation test (bottom panel). The red vertical line indicates the observed difference in the average number of contacts between symptomatic and asymptomatic cases, which lies at the far end of the null distribution.

From January 21 to March 6, 2020, there were 157 symptomatic cases and 30 asymptomatic cases in the Ningbo COVID-19 data [15]. These infected cases resulted in 2147 close contacts with them, of which 2001 exposures were caused by the symptomatic cases and 146 by the asymptomatic cases. The average number of close contacts by a symptomatic case was 13 and for an asymptomatic case was 5, and the difference is statistically significant with *P*<.001 from the permutation test. Figure 2 presents the histograms of the average numbers of contacts by the symptomatic and asymptomatic cases as well as the differences after the permutation (ie, under the null distribution) in the average numbers of contacts by the symptomatic and asymptomatic cases in the permutation test. The larger number of close contacts by the symptomatic cases may be due to the medical attention they received after they had the confirmation of a COVID-19 positive test.

### Results of the Fisher Exact Test

Under the Fisher exact test, we consider two scenarios: (1) to combine the numbers of symptomatic and asymptomatic cases as the total number of infected patients, leading to a 2×2 table; or (2) to separate them, leading to a 2×3 table, as shown in the primary analysis of close contacts section of Table 2.

From the results summarized in Table 2, we concluded that there was no significant difference in the transmission rates between the symptomatic and asymptomatic cases, either including or excluding the cases associated with the super-spreader. However, the tests excluding the cases associated with the super-spreader yielded larger *P* values: *P*=.84 when combining the numbers of symptomatic and asymptomatic cases, and *P*=.11 when separating them. As a result, there is no statistical evidence in the data to rule out the transmissibility of asymptotic carriers in comparison with symptomatic cases.

**Table 2:** Analysis of the transmission rates through close contacts by the symptomatic and asymptomatic cases of the coronavirus disease in Ningbo after removing all the cases associated with the super-spreader.

Analysis		Close contacts, n	Infected		Uninfected, n	*P* value	
			Symptomatic cases, n	Asymptomatic cases, n		Combined^a^	Separate^b^
**Primary analysis of close contacts by symptomatic and asymptomatic cases**						.84 (.37)^d^	.11 (.08)
	Symptomatic cases	1904 (97)^c^	79 (28)	15 (4)	1810 (65)		
	Asymptomatic cases	146	3	3	140		
	Total	2050 (97)	82 (28)	18 (4)	1950 (65)		
**Subgroup analysis by different relationships with infected cases**						<.001	<.001
	Family	268	37	10	221		
	Relatives	400	13	6	381		
	Friends	153	23	1	129		
	Coworkers	57	2	0	55		
	Medical	79	0	0	79		
	Others	1093	7	1	1085		
	Total	2050	82	18	1950		
**Subgroup analysis by different types of contact with infected cases**						<.001	<.001
	Daily activities	1048	69	14	965		
	Transportation	167	1	2	164		
	Medical contact	297	4	0	293		
	Other contact	538	8	2	528		
	Total	2050	82	18	1950		

^a^Combined means *P* values were obtained by pooling the numbers of symptomatic and asymptomatic cases together.

^b^Separate means *P* values were obtained by separating the numbers of symptomatic and asymptomatic cases.

^c^The numbers in the parentheses are associated with the super-spreader.

^d^*P* values in the parentheses were obtained when including the cases associated with the super-spreader.

### Estimation of the Odds Ratio

The estimated odds ratio, transmission rates, and their difference between symptomatic and asymptomatic cases as well as the corresponding 95% confidence intervals are all presented in Table 3. The odds of transmitting the coronavirus to a healthy individual by a symptomatic patient is 1.2 times more than that by an asymptomatic patient, which was not statistically significant as the 95% confidence interval covers one. Furthermore, as the 95% confidence intervals for the difference of transmission rates cover zero, we concluded that there is no difference in the transmissibility of the coronavirus through close contacts between symptomatic and asymptomatic cases, which is consistent with the findings using the Fisher exact tests.

The transmission rates under different relationships with the infected cases are significantly different with both *P* values<.001 whether combining the symptomatic and asymptomatic cases or not. With regard to different types of contact, the transmission rates are also significantly different with *P* values<.001. As expected, the more close contacts with the infected cases, the higher the likelihood of contracting the coronavirus.

**Table 3:** Primary analysis with the estimated rates and 95% CIs.

Variable	Odds ratio (95% CI)	Transmission rate of symptomatic cases, (95% CI)	Transmission rate of asymptomatic cases (95% CI)	Difference of transmission rates (95% CI)
With super-spreader cases	1.568 (0.679-3.620)	0.063 (0.053-0.075)	0.041 (0.017-0.091)	0.022 (–0.016 to 0.059)
Without super-spreader cases	1.212 (0.522-2.815)	0.049 (0.040-0.060)	0.041 (0.017-0.091)	0.008 (–0.029 to 0.046)

## Discussion

In summary, we provided a more in-depth analysis of the Ningbo COVID-19 data to examine the difference in the transmissibility of the coronavirus for symptomatic and asymptomatic patients. The conclusion remains the same, that there is no statistically significant difference in the transmissibility of the coronavirus between symptomatic and asymptomatic patients, but our evidence for no difference appears to be stronger with larger *P* values than the original analysis [15].

As the proportion of asymptomatic carriers in the total infected cases is considerably high [3-7], such findings are crucial to the public health and can help to guide the relevant government agencies on policy making about the asymptomatic cases.

However, our analysis only focuses on the data from the city of Ningbo, China, and the sample size is small. Therefore, the generalization of our findings to a larger and more diverse population is limited. More work is warranted to study the transmissibility of coronavirus by the asymptomatic coronavirus carriers.

## Abbreviations

COVID-19: coronavirus disease
RT-PCR: reverse transcriptase polymerase chain reaction
